# Vaccination

**Published:** 1804-04-01

**Authors:** 


### VACCINATION.

CITIZEN L. A. MONGENOT, Physician to the Hospital des Enfans Malades, at Paris, has lately published a Treatise on Vaccination, in which he has considered that interesting part of medicine in a point of view entirely new, by attempting to establish the theory of a truth now generally admitted. The existence of a fact may be sufficient for the vulgar, but a physical conviction will not be satisfactory to minds accustomed to develope causes; the man of science, as Citizen Mongenot judiciously remarks, wishes to obtain a moral conviction, which may submit the phenomena with which his senses are struck, to his understanding; it is not enough for him to see, he must comprehend. The author, therefore, makes it his business, not so much to ascertain a list of facts, as to concatenate a series of arguments — less to give calculations, than to furnish proofs; of course, he leaves to the Central Committee of Vaccination, of which he is a member, the care of ascertaining the credit due or not due to the enormous mass of facts of which it is the depositary. Faithful to the plan he has traced out, Cit. Mongenot begins by comparing the natural small-pox with the new specific. This parallel, established from day to day, and made with extreme precision, proves that vaccination proceeds in the same track as the small-pox, until the period when this latter occasions the general eruption; but as the general eruption, and the phenomena which accompany or follow it, do not constitute the essence of the small-pox; as it is only a series of symptoms which compromises the life of the subject infected with the variolous matter, without adding any thing to its future preservation from the same malady, it follows that vaccination and the small-pox exhibit a perfect analogy, as from the moment when they become dissimilar in their progress, this last has furnished its necessary period. Further, that it often stops of itself at this point, or, if it continues its further appearance, it is only to incur dangers more or less imminent, without offering any compensation in respect of future preservation. Here two questions suggest themselves: 1. Is it actually true that the general eruption and the maturatory fever do not constitute the preservative faculty of the natural or inoculated pock? When an assertion of this kind is confirmed by the testimonies in writing of Boerhaave, Van Swieten, Sydenham, Stoll, Rosen, Derosenstein, Huxham, La Condamine, and of all inoculators, even those who are hostile to inoculation, we may undoubtedly consider it as incontestible. We know besides, that inoculation has been considered as a benefit, only because it diminshes in general the pustular eruption, the only cause of danger, and pretty often secures from the secondary fever and from suppuration, the pustules only appearing in very small numbers. In fact, the symptoms which pertain to the resorption of the puriform matter of the pustules shew the existence of two effects essentially distinct in the small-pox; the first owing to the immediate action of the virus on the animal œconomy, and the second depending entirely on the inflammation and the suppuration of the pustules; but it is evident that the persons who have had the variolous fever without eruptions, have been always protected from the small-pox; it is manifest, therefore, that the eruption contributes nothing to ensure preservation. The second question, '*As it is proved* that the general eruption and the maturatory fever, do not constitute the preservative faculty, are they not merely a series of symptoms, the sole property of which is to compromise the life of the inoculated person? This second question is so closely connected with the former, that it is unnecessary to pursue it through its different ramifications. All the danger of the small-pox consisting in the critical eruption of the pustules, it was natural to seek to prevent, or at least to diminish it. Inoculation, in part, effected this end; but it was not a specific, for it entertained the focus of the malady, and perpetuated the contagious principle of it; it even sometimes developed a small, confluent pock, frequently mortal: the celebrated inoculator Gandoger had fatal experience of it on his own son. The true specific against the small-pox can only be that, which, by a virtue *sui generis*, was to destroy it compleatly. The great Boerhaave had a presentiment that one day we should be happy enough to find it. *Quale invenire posse, comparatio antidotorum et indoles hujus mali, faciunt sperare.* i. e. "*The possibility of such a dicovery is founded on the comparison of antidotes, and the character of the malady.*" Van Swieten expresses himself in a manner still more positive. "*Ex ante dictis constitit stimulum illum admodum exiguæ molis esse et tamen stupendas mutationes corpori humano, etiam sanissimo inducere, adeoq; sperari potest simile et remedium posse inveniri quod forte talis efficaciæ sit ut minima quoque ejus pars sufficiet enervando huic veneno. Ita forte invenietur aliquid quod venenum specificâ vi domare, sicque morbum in ipsis incunabulis*

*suffocare*

*suffocare valeat.*" Van. Sw. Variolæ, p. 54. i. e. *It ha therefore been proved that the mass of the contagious stimulus, in the smal -pox, is extremely small, and that, nevertheless, it can produce, even on the soundest body, stupendous alterations. There is, therefore, reason to hope for the discovery of a remedy perhaps so efficacious, that it will be necessary to employ only a very feeble dose to enervate this virus. In like manner it is possible to find a specific powerful enough to triumph over the small-pox, by stifling it as it were in the cradle.* We may further quote this passage of La Condamine: "Neither the eruption, nor the pustules, are essential to the natural or to the artificial small-pox; and perhaps a day will come that shall realise what Boerhaave and Lobb attempted; that is to say, to change the *exterior form* of this malady, without augmenting the danger. *Premier Memoire,* p. 90, &c. This specific, announced by Boerhaave, Lobb, Van Swieten, and so many others, is the cow-pock.

1. Because it exactly fulfils all the conditions required by those who have predicted it.

2. Because it is a preservative from the variolous or small-pock.

3. Because it prevents contagion, and is not contagious of itself.

4. Because it only produces a very slight malady.

5. Because its progress is identical to that of the inoculated small-pock.

And, 6. Because it does not occasion a general eruption equally terrible and useless.

Cit. Mongenot concludes by annexing an interesting list of cases of vaccination that have occurred within the sphere of his owh practice. In this part he throws out a challenge to detractors, antagonists, &c. that it will never be possible to infect the *preserved* subject with the variolic virus. He began his experiments upon his own son, the only one of his vaccinated patients that he submitted to the counter-experiment, by causing him to be inoculated with the small-pock. "It was useless," adds the author, "to repeat this fine experiment upon others; but I owed it to fathers of families to afford them such a guarantee as a father only could give."

